# Late-onset severe neutropenia in patients with relapsing remitting multiple sclerosis treated with ocrelizumab: case report and literature review

**DOI:** 10.3389/fnins.2025.1685209

**Published:** 2025-11-20

**Authors:** Catherine Tauro, Zeinab Awada, Radhika Malhotra, Asaff Harel

**Affiliations:** 1 Department of Neurology, Northwell Health, Donald and Barbara Zucker School of Medicine at Hofstra/Northwell, Hempstead, NY, United States; 2 Department of Emergency, Northwell Health, Donal and Barbara Zucker School of Medicine, Hempstead, NY, United States; 3 Northwell Comprehensive MS Center, Department of Neurology, Northwell Health, Donald and Barbara Zucker School of Medicine at Hofstra/Northwell, New York, NY, United States

**Keywords:** multiple sclerosis, ocrelizumab, late-onset neutropenia, autoimmune, case report

## Abstract

**Objective:**

To describe two cases of recurrent, delayed-onset severe neutropenia several months following ocrelizumab therapy in patients with relapsing–remitting multiple sclerosis (RRMS).

**Background:**

A rare adverse effect of ocrelizumab is neutropenia, with late-onset neutropenia (LON) occurring more rarely. Literature guiding management of transient recurrent neutropenia in the setting of anti-CD20 therapy is lacking.

**Methods:**

Case 1: 38-year-old female emergency physician with RRMS developed severe transient spontaneously resolving asymptomatic neutropenia 3 months after ocrelizumab infusion. Two years later, she developed severe symptomatic LON and required antibiotics and granulocyte colony-stimulating factor (GCSF). Ocrelizumab was held, patient switched to ozanimod, but neutropenia recurred. Due to concerns of MS progression, ocrelizumab was restarted after the patient transitioned to a telehealth setting, with no recurrence of neutropenia at one-year follow-up. Case 2: 35-year-old male emergency physician with RRMS developed severe transient spontaneously resolving asymptomatic neutropenia 3 months after ocrelizumab infusion. Ocrelizumab was resumed after absolute neutrophil count recovery. Two years later, he developed moderate symptomatic LON during a suspected viral illness. Ocrevus was discontinued at this point. A subsequent episode occurred 3 months later during confirmed rhinovirus infection, again resolving promptly.

**Conclusion:**

These cases highlight the unpredictable nature of recurrent LON with ocrelizumab and suggest the possibility of immune-mediated marrow suppression, potentially unmasked or worsened by infections, rather than direct drug toxicity, highlighting the need for clearer management guidelines.

## Introduction

Ocrelizumab is a humanized IgG-1 monoclonal anti-CD20 antibody approved for relapsing remitting multiple sclerosis. It exerts its therapeutic effect through the depletion of circulating immature and mature B cells. This decreases inflammation and destruction of myelin. A documented but rare adverse effect of ocrelizumab is neutropenia. The delayed onset of neutropenia after ocrelizumab administration and incident recurrence even when ocrelizumab is stopped is exemplified in this case series.

## Materials and methods

Case series with literature review performed.

### Case 1

A 38-year-old female emergency physician with a history of RRMS was diagnosed 3 years prior after developing abrupt onset of leg weakness, gait disturbance, and dizziness. Treatment with ocrelizumab was initiated several months after MS diagnosis and was well-tolerated, leading to clinical stability. Three months after her 2nd round of ocrelizumab, she was found to have severe asymptomatic neutropenia (ANC 0.13 K/μL). It resolved spontaneously to normal levels (ANC 1.44 K/μL) within 5 days, and the patient continued ocrelizumab and received the third infusion uneventfully after consultation with hematology. Five months later, ocrelizumab was temporarily halted due to pregnancy, then resumed post-delivery with the fourth infusion given around 15 months from ocrelizumab cessation. Four and a half months following the fifth dose, she developed severe symptomatic neutropenia, with an ANC of 0 K/μL associated with fevers, cough, sore throat, myalgias, night sweats, and malaise. The patient was admitted and treated empirically with cefepime and posaconazole. An extensive infectious work up did not reveal a specific pathogen. Hematology was consulted and suspected this to be secondary to ocrelizumab, though it was felt to be unusual for neutropenia to present symptomatically at a time when blood drug levels would be expected to be very low. The patient underwent an extensive work up for hematological etiologies of neutropenia, all of which was unrevealing, including a normal bone marrow biopsy that demonstrated no sign of blasts or acute leukemia. She was started on filgrastim, and the ANC normalized. Given the severity of her MS, it was deemed that the risks of delaying disease modifying therapy were too high, and she began taking ozanimod several weeks after normalization of ANC.

One and a half months later, she again developed neutropenia with a nadir of 0.85 K/μL. Given an absence of other etiologies, her neutropenia was once again attributed to ocrelizumab use, though this was nearly 8 months after her final ocrelizumab infusion. Ozanimod was stopped and filgrastim was restarted. Her levels eventually normalized 2 weeks later and ozanimod was restarted. She continued to receive ozanimod, and her ANC stabilized thereafter. During subsequent follow-ups, she exhibited gradual right lower extremity weakness over several months, concerning for MS progression. After a thorough discussion, she opted to restart ocrelizumab 1 year after the severe neutropenic episode with close monitoring of her ANC. Soon after restarting ocrelizumab treatment, she had also transitioned to a career in telehealth emergency medicine to reduce stress and infection risk. Her ANC has remained stable in the year since restarting ocrelizumab.

**Figure 1:**
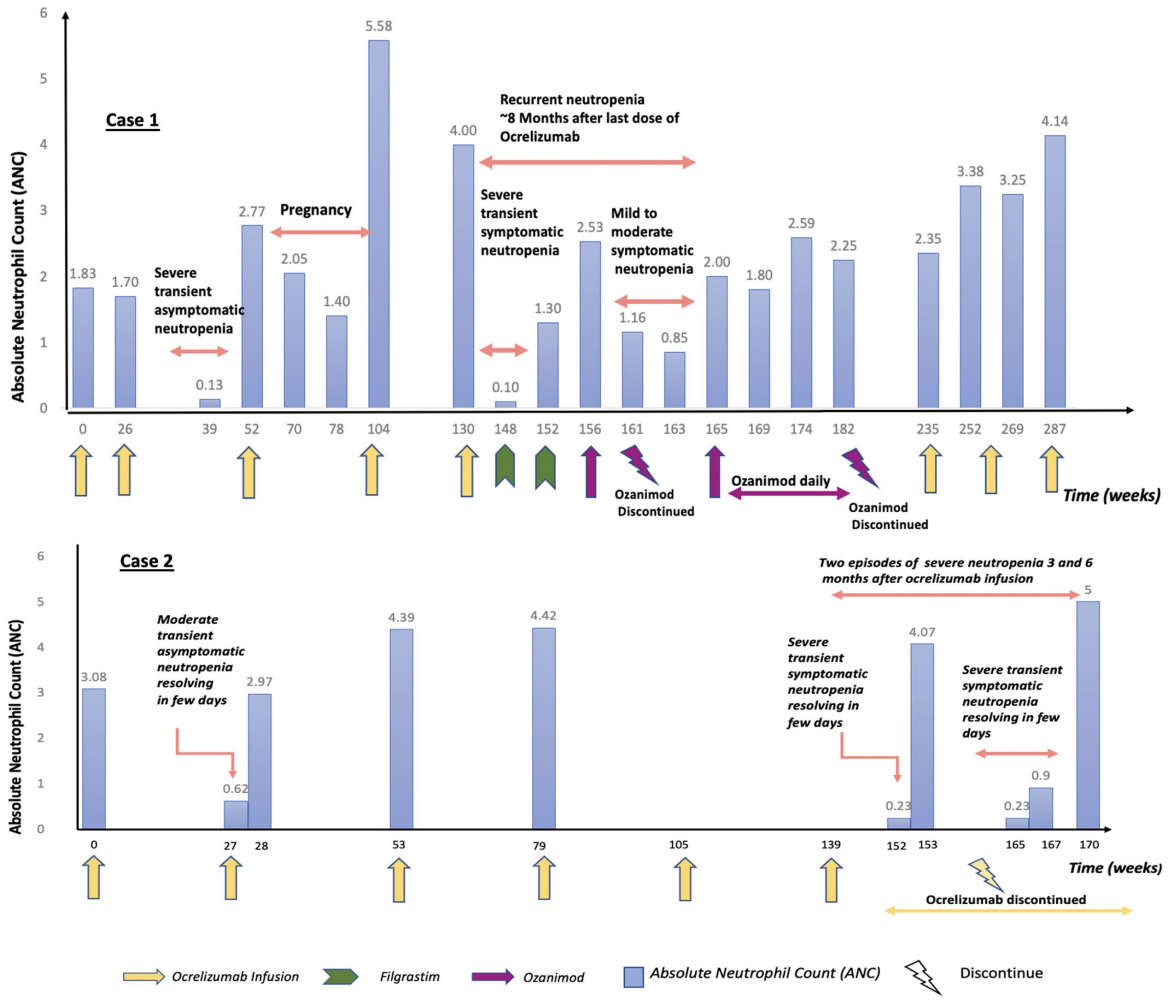
Timeline showing ANC change with ocrelizumab infusion in case 1 and case 2.

The detailed timeline is outlined in Figure 1.

### Case 2

A 35-year-old male emergency physician with a history of RRMS was diagnosed 10 years prior following a brainstem clinical attack. He was initially treated with interferon beta-1b for 1 year, after which he was transitioned to dimethyl fumarate. Five years later, he was switched to ocrelizumab. Two years after starting ocrelizumab, he developed leukopenia (WBC 2.71 K/μL) with moderate asymptomatic neutropenia (ANC 0.62 K/μL). This episode was transient and resolved spontaneously within 7 days. Given the absence of symptoms and the resolution of neutropenia, ocrelizumab was resumed after hematology clearance. Two years later, and 3 months following an ocrelizumab infusion, he was admitted with an upper respiratory tract infection-like illness and was found to be profoundly neutropenic (ANC 0.23 K/μL). Infectious workup did not reveal a specific pathogen. A peripheral blood smear showed neutropenia without the presence of leukoblasts, but with a considerable number of activated and atypical monocytes, consistent with a viral infection. The neutrophil count spontaneously normalized 3 days later (ANC 3.36 K/μL). The patient had another episode of symptomatic neutropenia 3 months later despite no further ocrelizumab treatment (6 months after his last infusion). He again experienced upper respiratory tract infection symptoms. His ANC was again low at 0.23 K/μL and he was found to be positive for rhinovirus on nasal swab. The ANC improved the next day to 0.99 K/μL, and the patient was discharged. One month later, the ANC normalized.

The detailed timeline is outlined in Figure 1.

## Discussion

LON is characterized by an absolute neutrophil count below 1.5 K/μL occurring more than 4 weeks after the last dose of treatment in patients with previously normal neutrophil counts and no other identifiable causes. While it was initially unexpected that CD20 targeting therapies would cause LON, it has been reported in patients receiving both ocrelizumab and rituximab, two monoclonal anti-CD20 antibodies that share a similar mechanism of action (Marrodan et al., 2021). Although data on LON associated with ocrelizumab are limited, its incidence following rituximab administration ranges from 3 to 27% (Wolach et al., 2010). Most cases are reported to be mild or self-limiting with an average time to diagnosis ranging between 90 and 150 days in a rheumatology cohort (Breuer et al., 2014).

Neutropenia has been reported in clinical trials of ocrelizumab; however, the majority of cases were transient, non-severe, and did not require discontinuation of treatment. In the ORATORIO trial, decreased neutrophil counts were reported in 13% of patients treated with Ocrevus, compared to 10% in the placebo group. In most cases, this decrease was not recurrent and neutrophil count ranged between 1.0–1.5 K/μL. Overall, 1% of ocrelizumab-treated patients in the trial experienced neutrophil counts below 1.0 K/μL, and these episodes were not associated with any documented infections (Ocrevus (Ocrelizumab) Full Prescribing Information, 2017). Subsequent safety analysis that included data from the controlled treatment and open-label extension (OLE) periods of the phase 2, 3 and 3b ocrelizumab trials showed that approximately 4.4% of patients in the OPERA trials and 4.6% of patients in the ORATORIO trial experienced neutropenia defined as ANC < 1.5 K/μL. Most episodes were isolated, not sustained or replicated, and did not necessitate cessation of ocrelizumab therapy (Hauser et al., 2021).

The onset time for delayed neutropenia has exhibited variability across reported cases. One small case series demonstrated LON can arise around 4 months after rituximab infusion, lasting about 5 days (Rigal et al., 2020). Other reports with ocrelizumab have shown late-onset neutropenia following the last administered dose of ocrelizumab to manifest between 2.5 to 10 months (Rauniyar et al., 2022; Alabdulqader et al., 2024; Auer et al., 2020; Zanetta et al., 2020). In another case series and systematic review focusing on neutropenia complicating anti-CD20 treatment, the average onset of neutropenia was observed after two treatment cycles, with delayed neutropenia occurring approximately 90 days after a treatment (Rossi et al., 2022). Most patients in the cohort experienced symptomatic severe neutropenia requiring antibiotics and G-CSF, and nearly a 20% recurrence rate was observed in patients continuing anti-CD20 therapy (Rossi et al., 2022). These results were different from the OPERA & ORATORIO studies that showed transient non-severe neutropenia associated with ocrelizumab use.

Neutropenic episodes in patients receiving ocrelizumab may occur intermittently, potentially triggered by bacterial or viral infections. A large cohort study of patients with MS and neuromyelitis optica spectrum disorders (NMOSD) found that neutropenia associated with B-cell–depleting therapy although rare, was often clinically significant, and infections were possible triggers (Cerqueira et al., 2025). In our report, recurrent delayed-onset neutropenia was observed in two patients, both of whom were emergency department physicians with frequent occupational exposure to infectious pathogens. This high-risk environment may have played a contributory role in the recurrence of neutropenic episodes in the setting of ongoing ocrelizumab therapy. Consequently, it is important to investigate potential causes of acquired neutropenia in ocrelizumab-treated patients, such as viral infections, while acknowledging that the underlying mechanism may involve impaired immune regulation rather than being solely attributable to infection.

Recent long-term data support the overall favorable safety profile of ocrelizumab. Over more than a decade of continuous use, serious infection rates have remained low and stable, with no new safety signals identified (Guerra et al., 2025). Although ocrelizumab modulates both humoral and cellular immune responses, these immunologic effects do not appear to translate into an increased long-term infection risk despite reductions in immunoglobulin levels and peripheral lymphocyte subsets during prolonged ocrelizumab treatment (Waldrop et al., 2025).

The mechanism by which ocrelizumab and rituximab induce LON remains unclear, although various hypotheses have been proposed. One suggested mechanism involves antibody-mediated destruction through immunological pathways, leading to the production of autoantibodies against neutrophils. This process could occur directly or via the FAS/FAS ligand pathway, which induces neutrophil apoptosis (Baker et al., 2024). However, neutropenia does not appear to be mediated by specific antineutrophil antibodies, as the CD20 receptor is not expressed on granulocytes or their progenitor cells. Alternatively, genetic factors, such as specific polymorphisms in the immunoglobulin G Fc receptor (FCγ IIIa 158 V/F), could predispose certain individuals to developing neutropenia as an adverse effect (Tesfa and Palmblad, 2011). Nevertheless, both these mechanisms seem inconsistent with our cases, as neutropenia persisted at a time when ocrelizumab drug levels were likely negligible. Another proposed mechanism involves a direct impact on the bone marrow, causing an imbalance between granulopoiesis and lymphopoiesis. Increased blood levels of B-cell activating factor (BAFF) have been detected at the onset of LON (Terrier et al., 2007). BAFF may inhibit promyelocyte maturation during B-cell lineage recovery, an effect that could persist even after drug clearance (Terrier et al., 2007). In both of our cases, severe neutropenia recurred well after cessation of ocrelizumab therapy at a time when drug levels would be expected to be minimal or absent. This suggests that LON could result from a subsequent immune dysregulation triggered by B cell depletion, with downstream effects persisting beyond the pharmacologic activity of the drug.

The incomplete understanding of the mechanism underlying LON contributes to the absence of clear recommendations for its management. While granulocyte-colony stimulating factor (GCSF) has been reported to potentially cause flare-ups of MS (Openshaw et al., 2000), other series have described its use without exacerbating MS (Rossi et al., 2022), and it may offer therapeutic benefit by accelerating recovery in patients experiencing LON due to ocrelizumab. Continuation of ocrelizumab after an episode of LON is not contraindicated, but the risk of recurrence remains uncertain. Data from a large retrospective cohort of 738 patients treated with rituximab for nephrological and systemic autoimmune diseases showed a cumulative incidence of recurrent LON of 11.5% (95% CI: 5.6–22.6) at 1 year, 23.4% (95% CI: 13.8–38.2) at 2 years, and 30.4% (95% CI: 16.9–50.9) at 5 years (Zonozi et al., 2021). Similarly, recurrent episodes of LON were reported in a case series involving ocrelizumab, with recurrences happening soon after the first episode (within 7 to 40 weeks) (Beigneux et al., 2024). These episodes were symptomatic, with extremely low neutrophil counts (nadir <50/mm^3^), but the clinical course improved quickly and outcome was favorable in all cases.

In both of our cases, the initial episodes of neutropenia were transient and asymptomatic, allowing for the continuation of ocrelizumab after appropriate evaluation and resolution. Both patients subsequently experienced recurrent episodes of more severe neutropenia. However, despite shared features, the clinical courses differed. The first patient exhibited a prolonged episode of symptomatic grade 4 neutropenia that required G-CSF support. While infection was considered a potential contributing factor, no definitive source was identified. In contrast, the second patient experienced recurrent episodes of neutropenia in the setting of clearly documented infections. These episodes were profound but resolved rapidly within days and without intervention, in contrast to the more sustained and treatment-requiring course observed in the first case. Notably, the immunoglobulin levels of both patients remained within normal limits. In total, this pattern suggests the possibility of an impact on granulopoiesis, potentially unmasked or exacerbated by infection-related neutrophil cell death. Notably, both patients were emergency physicians with frequent occupational exposure to infectious pathogens, which may have contributed to the timing and recurrence of neutropenic episodes. Re-exposure to ocrelizumab in the first patient 1 year after the severe episode did not lead to further recurrence, suggesting that additional random factors—such as intercurrent viral illness— may be required to trigger neutropenia in this susceptible individual. However, it is also interesting to note that she transitioned to a telehealth setting shortly after restarting ocrelizumab, possibly reducing overall infection exposure. These cases reinforce the notion that LON associated with ocrelizumab is a multifactorial process and a potentially underrecognized complication.

## Conclusion

Although further studies are warranted, our cases contribute to the emerging evidence of delayed and recurrent neutropenia associated with ocrelizumab. It also highlights the importance of surveillance to promptly detect and address severe neutropenia and possible infections. Routine frequent blood sampling following ocrelizumab administration may be indicated to detect asymptomatic occurrence of LON. This unpredictable and rare adverse event adds to the understanding of LON with ocrelizumab and may help in developing further guidelines for management.

## Data Availability

The original contributions presented in the study are included in the article/supplementary material, further inquiries can be directed to the corresponding author.
